# Intensity-modulated radiotherapy combined with albumin-bound paclitaxel and platinum-based chemotherapy for locoregional recurrent cervical cancer: a single-institutional phase II investigator-initiated nonrandomized clinical trial

**DOI:** 10.1007/s00432-026-06555-0

**Published:** 2026-07-28

**Authors:** Chi Fang, Suping Liu, Jie Xia, Xiaohua Wu, Guihao Ke, Jun Zhu

**Affiliations:** 1 Department of Gynecologic Oncology, Fudan University Shanghai Cancer Center, 270 Dong-An Road, Shanghai, 200032 China; 2 Department of Oncology, Shanghai Medical College, Fudan University, Shanghai, China; 3 Department of Digestive Endoscopic Center, Shanghai Jiao Tong University Affiliated Sixth People’s Hospital, Shanghai, China

**Keywords:** Cervical cancer, Locoregional recurrence, Radiotherapy, Chemotherapy, Survival

## Abstract

**Objective:**

To prospectively explore efficacy and safety of radiotherapy combined with albumin-bound paclitaxel and platinum-based chemotherapy for locoregional recurrent cervical cancer (CC).

**Methods:**

Patients with locoregional recurrent CC were enrolled in the analysis. The enrolled patients received radiotherapy and albumin-bound paclitaxel and platinum-based chemotherapy. The primary endpoint was overall survival (OS). Secondary endpoints were progression-free survival (PFS), objective response rate (ORR), and safety.

**Results:**

99 patients were enrolled. For all patients, the 3-year PFS and 3-year OS were 48.5% and 58.6%. 17.2% of patients achieved CR and 67.7% of patients achieved PR. The ORR was 84.8%. Moreover, 10.1% of patients were confirmed SD. The DCR was 94.9%.

**Conclusion:**

Locoregional recurrent CC could benefit from radiotherapy combined with albumin-bound paclitaxel and platinum-based chemotherapy.

## Introduction

Cervical cancer (CC) is the fourth most prevalent cancer among women on a global scale (Salani et al. 2024). The evolution of modern diagnostic and treatment modalities has resulted in a significant decrease in mortality and recurrence rates (Zhang et al. 2022). Nevertheless, recurrence persists as a critical issue, affecting 29–38% of treated patients. Early-stage cervical cancer also presents a recurrence rate of 10–15% (Velden et al. 2019). While recurrence patterns in cervical cancer can differ, approximately 75% of recurrent lesions are localized to the pelvic cavity (Boussios et al. 2016).

Following the 1999 NCI clinical alert, cisplatin-based concurrent chemoradiation has become the standard treatment for cervical cancer, owing to the potent radiosensitizing property of cisplatin (Morris et al. 1999). Phase I clinical trials have confirmed that simultaneous radiotherapy with paclitaxel and cisplatin is safe and feasible for cervical cancer, and the synergistic effect of the two enhances local control (Prasad et al. 2009). In Phase II clinical trials, the combination of albumin-bound paclitaxel and platinum was used for recurrent/advanced cervical cancer, confirming the anti-tumor advantages (Li et al. 2017). Accumulating preclinical and clinical evidence supports the feasibility of cisplatin plus nab-paclitaxel combined with radiotherapy for cervical cancer. Despite these efforts, the therapeutic outcomes for recurrent and metastatic cervical cancer remain unsatisfactory. The median overall survival (OS) with systemic chemotherapy is documented at 13.3 months, and the addition of bevacizumab only marginally increases this to 16.8 months (Tewari et al. 2017). These findings underscore the urgent need for more effective treatment protocols.

Recent years have witnessed rapid advancements in radiotherapy technology. The refinement of external beam radiation therapy (EBRT) and the incorporation of image-guided radiotherapy (IGRT) have substantially improved target conformity while reducing radiation exposure to organs at risk (OARs) (Forrest et al. 2012; Webster et al. 2020). These technological advances have expanded treatment options for previously challenging scenarios, including recurrent and metastatic cervical cancer. Radical radiotherapy with concurrent chemotherapy remains the cornerstone treatment of locally advanced cervical cancer.

A retrospective cohort study demonstrated that chemoradiation with simultaneous integrated boost to positive lymph nodes achieved favorable oncologic outcomes with manageable late toxicities in patients with para-aortic metastatic cervical cancer (Gao et al. 2024). Moreover, a retrospective review of 121 patients with recurrent cervical cancer post-surgery indicated that chemoradiotherapy, similar to salvage surgery, was the predominant treatment, yielding a 5-year survival rate of 22.3% (Qiu et al. 2012). Collectively, these findings underscore the therapeutic potential of radiotherapy in the management of recurrent cervical cancer. Motivated by the growing body of evidence, we propose to explore the efficacy of radiotherapy combined with albumin-bound paclitaxel and platinum-based chemotherapy on locoregional recurrent cervical cancer.

## Materials and methods

### Study design and patient selection

This phase II clinical study was designed as a prospective, single-arm, single-center, exploratory investigation to evaluate the efficacy and safety of radiotherapy with albumin-bound paclitaxel and platinum-based chemotherapy for patients with locoregional recurrent cervical cancer (CC). The study protocol and all amendments were reviewed and approved by the institutional ethics committee of Fudan University Shanghai Cancer Center (No.2004216-1-2006). All patients provided written informed consent prior to enrollment.

Patients were included if they had: locoregional recurrent cervical cancer; oligometastatic disease suitable for radiotherapy assessed by radiation oncologist; oligometastatic disease outside previous radiation field; pathological diagnosis of squamous cell carcinoma, adenocarcinoma, or adenosquamous cell carcinoma; an Eastern Cooperative Oncology Group performance status (ECOG PS) of 0–2; an expected survival of at least 3 months; and measurable disease based on the Response Evaluation Criteria in Solid Tumors (RECIST) version 1.1 (Eisenhauer et al. 2009).

Exclusion criteria included: the presence of other active malignancies; pregnancy or breastfeeding; conditions that impair oral medication use (e.g., difficulty swallowing, chronic diarrhea, intestinal obstruction); any bleeding event graded ≥ 4 by the National Cancer Institute Common Terminology Criteria for Adverse Events (NCI-CTCAE) (Grover et al. 2018) within 4 weeks before screening; uncontrolled hypertension; history of unstable angina or abnormal coagulation function; arterial or venous thrombosis within 1 year prior to screening; and a history of immunodeficiency.

### Procedures and assessment

Between September 2020 and September 2023, patients were enrolled at the Fudan University Shanghai Cancer Center. Participants received intensity-modulated radiotherapy (IMRT) planned with CT guidance. Target delineation was based on the Radiation Therapy Oncology Group Consensus Guideline 2008 (Small et al. 2008). Treatment plans utilized a megavoltage simulator with 6 MV photon energy, optimized to ensure that 95% of the planning target volume (PTV) received the prescribed dose.

Chemotherapy adopted albumin-bound paclitaxel and platinum-based regimen, with 4 cycles. The predominant chemotherapy regimens were TP (albumin-bound paclitaxel, 260 mg/m^2^, and cisplatin, 70–75 mg/m^2^, Q3W) “or” TC (albumin-bound paclitaxel, 260 mg/m^2^, and carboplatin, AUC 5, Q3W). If the patient cannot tolerate cisplatin, carboplatin was used instead. Chemotherapy and radiotherapy were carried out simultaneously, and 99 patients completed 4 cycles of chemotherapy.

Treatment responses were assessed by the study investigators using RECIST version 1.1 criteria, with computed tomography (CT) or magnetic resonance imaging (MRI) performed at baseline, and 1 month after the completion of radiotherapy. Patients underwent a follow-up examination every three months for the first two years, and then every six months for the next 5-years.

The study evaluated progression-free survival (PFS), overall survival (OS), complete response (CR), partial response (PR), objective response rate (ORR), disease control rate (DCR), and safety. The ORR was calculated as the sum of CR and PR, while the DCR included CR, PR, and stable disease (SD).

### Statistical analysis

This exploratory phase II clinical study aims to assess the potential for increasing the 3-year survival rate of locoregional recurrent cervical cancer. Existing data suggest a 3-year survival rate of 30% following such treatment (Tewari et al. 2014). The study's goal is to achieve a 3-year survival rate of 45%. The study was designed with an alpha value of 0.05, a power of 0.8, and a beta of 0.2. Sample size calculations using PASS software indicated a requirement of 90 cases. To account for a 10% dropout rate, the planned sample size was adjusted to 99 cases.

The 95% confidence interval (CI) was determined using the Wilson method. Kaplan–Meier methods were utilized to estimate progression-free survival (PFS) and overall survival (OS). Survival curves were compared between different groups using the log-rank test. A p-value of less than 0.05 was considered statistically significant. Data analysis was performed using GraphPad Prism 9.

## Results

### Patient clinicopathological characteristics

**Fig. 1:**
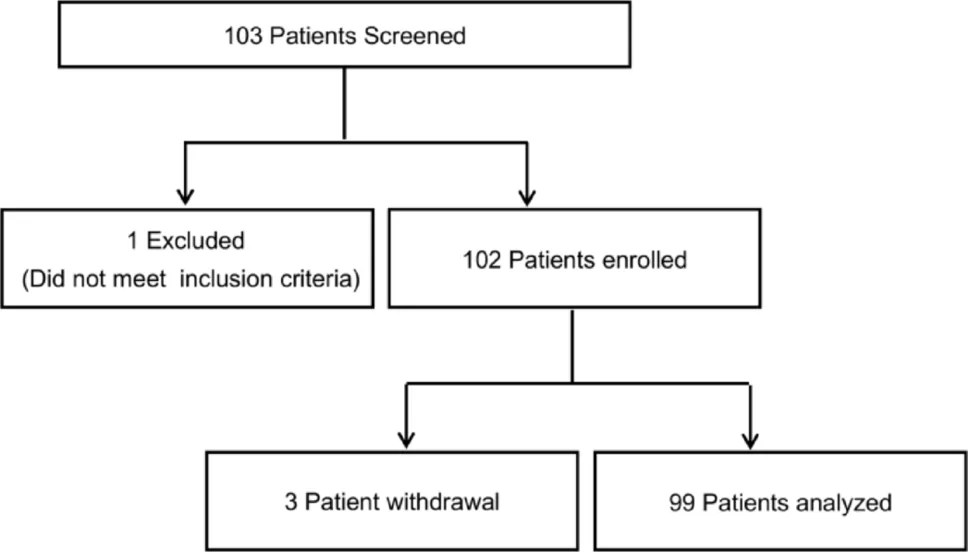
The flowchart of the trial

Of the 103 patients screened, 99 patients were analyzed in the study. A flow diagram depicting the consent and enrollment process is shown in Fig. 1. Summary statistics for the baseline characteristics of the patients were provided in Table 1. The median age was 55 years (range: 23–69 years). Histological findings indicated that 90.9% (90/99) of patients had squamous cell carcinoma, 5.1% (5/99) had adenocarcinoma, and 4.0% (4/99) had other histological types. The majority of patients (97) had an ECOG PS score of 0, with two patients having an ECOG PS score of 1. The majority of recurrences were localized to the pelvic cavity (76.8%), whereas 15.2% of patients presented with retroperitoneal lymph node involvement and 8.1% had recurrences both within and outside the pelvic cavity.

**Table 1:** Baseline patient characteristics

Characteristic	N (%)
Age (years, median)	55
*Histology*	
Squamous cell carcinoma	90(90.9)
Adenocarcinoma	5 (5.1)
Adenosquamous cell carcinoma	4 (4.0)
*ECOG*	
0	97(98.0)
1	2(2.0)
*Frequency of recurrences*	
1	99 (100.0)
> 1	0 (0.0)
*Previous systemic chemotherapy*	
Yes	0 (0.0)
No	99 (100.0)
*Sites of recurrence*	
1. Within the pelvic cavity:	76 (76.8)
*Vaginal stump*	45 (45.5)
*Pelvic mass*	5 (5.1)
*Pelvic lymph nodes*	26 (26.3)
2. outside the pelvic cavity:	
*Retroperitoneal lymph nodes*	15 (15.2)
3.1 + 2	8 (8.1)
*Lymph node metastasis*	
Yes	49 (49.5)
No	50 (50.5)
*Number of recurrent sites*	
≤ 5	99 (100.0)
> 5	0 (0.0)
Reirradiation	0 (0.0)
Prior chemotherapy lines	0 (0.0)

All patients received intensity-modulated radiotherapy (IMRT) planned with CT, delivered at radical radiation doses of 5880 cGy/28fx for Gross tumor volume. Gross tumor volume (GTV) delineation was based on pre-treatment enhanced CT or MRI. The clinical target volume (CTV) is detailed in Table 2, and was given doses of 5040 cGy/28fx. Patients with pelvic recurrence received pelvic field radiotherapy, which included the iliopsoas, external iliac, internal iliac, sacral anterior regions, and drainage areas of the obturator lymph nodes, as well as the vaginal stump, proximal vagina, paravaginal tissue, and parametrial tissue. For patients with recurrence outside the pelvic cavity, extended field radiotherapy was administered, covering pelvic field and periaortic lymph node drainage areas. A 5–7 mm margin expansion of the CTV was used to define the planning target volume (PTV). Monthly follow-up assessments were conducted for the first six months following radiotherapy.

**Table 2:** Clinical target volume

Location	CTV
1. Within the pelvic cavity	Pelvic field
2. Retroperitoneal lymph nodes	Extended field
3. 1 + 2	Extended field

*Pelvic field*: the iliopsoas, external iliac, internal iliac, sacral anterior regions, and the drainage areas of the obturator lymph nodes, as well as the vaginal stump, proximal vagina, paravaginal tissue, and parametrial tissue

*Extended field*: Pelvic field + periaortic lymph node drainage area

### Survival and recurrence analysis

**Fig. 2:**
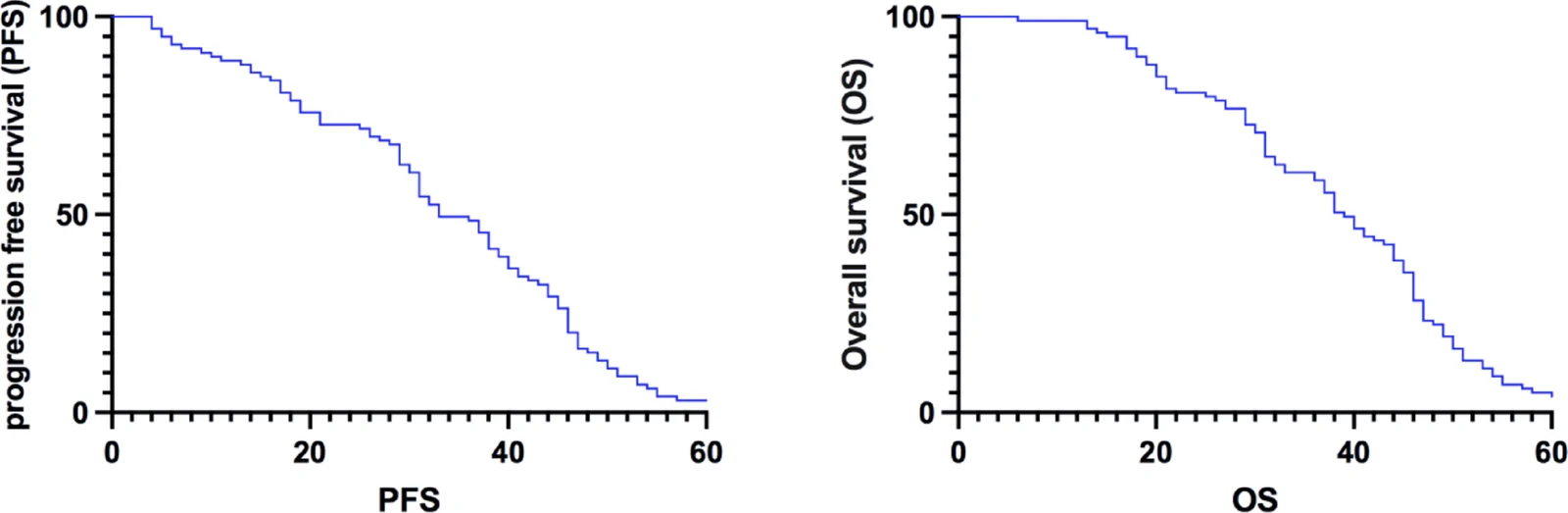
Progression-free survival (PFS) and overall survival (OS)

As of the observation cutoff date in September 2025, the median follow-up duration was 39 months. The 3-year progression-free survival (PFS) and overall survival (OS) were 48.5% and 58.6%, respectively (Fig. 2). These outcomes mark a substantial improvement over prior reports, which documented a 3-year survival rate of 41% for recurrent cervical cancer (Howlett, et al. 2024).

In the follow-up period, treatment response was evaluated in 99 patients. Complete response (CR) was achieved in 17 patients (17.2%), and partial response (PR) was observed in 67 patients (67.7%). The objective response rate (ORR) was 84.8% (95% CI: 76.5–90.6). Stable disease (SD) was confirmed in 10 patients (10.1%), and progressive disease (PD) was confirmed in 5 patients (5.1%). The disease control rate (DCR) was 94.9% (95% CI: 88.6–98.1) (Table 3).

**Table 3:** Overall tumor response in patients

Parameter	N (%)
Complete response (CR)	17 (17.2)
Partial response (PR)	67 (67.7)
Stable disease (SD)	10 (10.1)
Progressive disease (PD)	5 (5.1)
ORR (CR + PR), 95%CI	84 (84.8), 76.5–90.6
DCR (CR + PR + SD), 95%CI	94 (94.9), 88.6–98.1

Data are presented as N (%) unless otherwise specified. Responses were assessed in accordance with RECIST version 1.1. Only confirmed responses were included

ORR, overall response rate; DCR, disease control rate; CI, confidence interval

Our study indicates that the combination of radiotherapy and albumin-bound paclitaxel and platinum-based chemotherapy achieves satisfactory local control and significantly influences PFS and OS in patients with locoregional recurrent cervical cancer.

### Toxicity and safety analyses

Treatment-related adverse events (TRAEs) of grade ≥ 3 occurred in 7 of 99 (7.1%) patients. The most common TRAE of grade ≥ 3 was hematological toxicity. The most TRAEs were acute toxicities, and the common TRAEs were alopecia, hematological toxicity including decreased neutrophil count, decreased WBC count, decreased platelet count, and anemia (Table 4).

**Table 4:** Adverse events

Adverse events	Grade 1 + 2 (N)	Grade 3 + 4 (N)
Hematological toxicity	75 (75.8%)	5 (5.1%)
Nausea	28 (28.3%)	2 (2.0%)
Diarrhea	19 (19.2%)	0 (0.0)
Neuropathy	62 (62.6%)	0 (0.0)
Alopecia	99 (100.0%)	0 (0.0)
Fistula formation	0 (0.0)	0 (0.0)
Bowel obstruction	0 (0.0)	0 (0.0)
Allergy	0 (0.0)	0 (0.0)
Rash	0 (0.0)	0 (0.0)
Urinary tract infection	12(12.1%)	0 (0.0)
Late proctitis	4 (4.0%)	0 (0.0)

Late adverse effects were mild proctitis, which occurred in 4 of 99 (4.0%) patients.

## Discussion

Cervical cancer (CC) represents a substantial global health burden in gynecologic oncology, affecting millions worldwide (Ferrari and Giannini 2024). In China, it ranks as the sixth leading cause of cancer-related mortality, accounting for 16% of global CC deaths (Xiang et al. 2025). Despite advances in primary treatment, a considerable proportion of patients experience disease relapse. Patients with persistent, recurrent, or metastatic CC have limited therapeutic options and face a dismal prognosis, with a 5-year overall survival (OS) rate of less than 20% (Gopu et al. 2021; Lei et al. 2024). The development of novel therapeutic strategies for recurrent and metastatic cervical cancer is of paramount importance.

Pelvic exenteration (PE) remains the only curative surgical option for highly selected patients with isolated central pelvic recurrence, no lymph node/distant metastasis, and prior radiotherapy. In recurrent/persistent cervical cancer, PE achieves 3‑year OS of 29–61% and 5‑year OS of 30–49% (Stanca et al. 2022; Glane et al. 2022). Critically, patients with lymph node metastasis are routinely excluded from curative PE; only 20–33% of PE candidates are node‑negative, and nodal positivity confers significantly worse survival. For patients without lymph node metastasis (N0), concurrent chemoradiation yields ORR of 75–90%, 3‑year PFS of 50–60%, and 3‑year OS of 60–75%, which is comparable to PE but avoids major surgical morbidity. Importantly, in patients with lymph node metastasis (N +), who are ineligible for curative PE, concurrent chemoradiation still achieves ORR of 65%–80%, 3‑year PFS of 35%–45%, and 3‑year OS of 40–55%, representing a valuable curative‑intent option for this high‑risk population (Bonala et al. 2025; Lee et al. 2022).

Prior studies have documented the efficacy of radiotherapy in treating postoperative recurrence in cervical cancer (CC). In a cohort of 130 patients, radiotherapy achieved a 5-year disease-specific survival rate of 40.2%, with further analysis revealing 5-year disease-free survival rates of 55.4% for vaginal recurrence and 12.5% for pelvic lymph node recurrence. These results highlight the potential benefits of radiotherapy in recurrent CC, particularly for vaginal and pelvic lymph node involvement (Jain et al. 2007). Our prospective investigation focused on the efficacy and safety of radiotherapy combined with albumin-bound paclitaxel and platinum-based chemotherapy for locoregional recurrent cervical cancer. We observed that 17.2% of patients achieved complete response (CR), 67.7% achieved partial response (PR), and 10.1% achieved stable disease (SD). The objective response rate (ORR) stood at 84.8%, while the disease control rate (DCR) reached 94.9%. For patients with local recurrence, the 3-year progression-free survival (PFS) and overall survival (OS) rates were 48.5% and 58.6%, respectively. These figures represent a marked improvement over the previously reported 3-year survival rate of 41% for recurrent cervical cancer (Howlett, et al. 2024). Collectively, our findings underscore the significant therapeutic advantages of radiotherapy and chemotherapy in locoregional recurrent CC.

Investigations into the efficacy of combining radiotherapy with chemotherapy for recurrent and metastatic cervical cancer (CC) remain sparse. Thomas et al. reported outcomes for 41 patients with recurrent cervical cancer confined to the pelvis or involving pelvic and para-aortic lymph nodes following surgery. The treatment regimen consisted of 5-fluorouracil (5-FU) at 1.5 g/m^2^, succeeded by radiotherapy at 160 cGy per fraction, administered twice daily to a total dose of 5280 cGy over 33 fractions. Ten patients received additional chemotherapy with mitomycin C at 6 mg/m^2^ for 1 or 2 courses. Complete remission was documented in 58% (23/40) of patients, with 5 experiencing recurrence. At follow-up, 18 patients (45%) remained tumor-free, with a median survival duration of 57 months (range, 3–113 months) (Thomas et al. 1993). Maneo et al. described 35 patients with recurrent cervical cancer post-surgery treated with radiotherapy at 50–70 Gy, concurrent with 5-FU at 1000 mg/m^2^ on days 1–4 and carboplatin at 75 mg/m^2^ on days 1–4 for three courses. The treatment completion rate stood at 80%, with a median follow-up of 27 months. The 2-year and 3-year survival rates were 44% and 25%, respectively, and the median survival period was 21 months. Grade 3–4 adverse reactions were observed in 11 patients (31%), and late-stage reactions occurred in 5 patients (14%). Tumor-free survival was achieved in 37% (13 cases), tumor-bearing survival in 11% (4 cases), and 52% (18 cases) succumbed to disease progression (Maneo et al. 1999). Our advocacy for radiotherapy in recurrent CC is not the first of its kind. We have previously published studies on patients with recurrent or metastatic cervical cancer treated with three-dimensional conformal radiotherapy or intensity-modulated radiotherapy, demonstrating a 3-year survival rate of 56.1% and a local control rate of 59.7%. The 3-year progression-free and disease-free survival rates were 65.3% and 64.3%, respectively (Liu et al. 2012).

Recent clinical trials have positioned immunotherapy as a transformative treatment option for cervical cancer (CC), particularly in the recurrent or advanced disease setting. KEYNOTE-A10 has shown the effectiveness and safety of immunotherapy for locally advanced, unresectable and metastatic solid tumors refractory to therapy. KEYNOTE-826 phase III trial has been instrumental in establishing immunotherapy as a first-line standard therapy, demonstrating superior progression-free survival (PFS) and overall survival (OS) outcomes compared to chemotherapy alone. These results emphasize the significant potential of immunotherapy and advocate for its broader application in clinical practice (Monk et al. 2023a). The BEATcc trial further corroborated the efficacy of immunotherapy in metastatic, persistent, or recurrent CC, showing marked improvements in PFS and OS with atezolizumab compared to chemotherapy and bevacizumab alone (Oaknin et al. 2024). Conversely, the CALLA trial, which explored the addition of durvalumab to concurrent chemoradiotherapy (CCRT) for locally advanced CC, did not achieve its primary endpoints of enhanced PFS or OS (Monk et al. 2023b). This finding highlights the necessity for further investigation to define the precise role of immunotherapy in the comprehensive treatment strategy for cervical cancer. At the initiation of this study design (2020), the NCCN guidelines recommended a standard protocol of platinum based combination with paclitaxel ± bevacizumab without PD-1 inhibitor (Abu-Rustum, et al. 2020). As of 2026, based on positive results from KEYNOTE-826, NCCN guidelines have listed pembrolizumab/atezolizumab combined with chemotherapy ± bevacizumab as the first-line preferred option for patients with PD-L1 CPS ≥ 1 (category 1 evidence) (Abu-Rustum et al. 2025).

This study represents a novel prospective evaluation of the effects of radiotherapy and chemotherapy on prognosis and toxicity in recurrent cervical cancer (CC). Our findings provide valuable insights and establish a robust foundation for the development of effective therapeutic approaches for recurrent CC. However, as this study is not a randomized trial, the findings should be interpreted with caution. Future prospective studies with extended follow-up periods are warranted to validate the clinical efficacy of the proposed treatment strategies. It is also important to note that the relatively short follow-up period in this study may introduce bias in the results, emphasizing the need for longer-term follow-up in subsequent research.

## Data Availability

No datasets were generated or analysed during the current study.

## Abbreviations

CC: Cervical cancer
PFS: Progression-free survival
OS: Overall survival
IMRT: Intensity-modulated radiotherapy
CR: Complete response
PR: Partial response
ORR: Objective response rate
DCR: Disease control rate
SD: Stable disease
GTV: Gross tumor volume
CTV: Clinical target volume
PTV: Planning target volume
